# Jean-François Morot-Gaudry (1943–2024): A Life Devoted to Plant Science, Leadership, and Humanity

**DOI:** 10.3390/plants14243711

**Published:** 2025-12-05

**Authors:** Jean-François Briat, Jean-Bernard Cliquet, Michel Delseny, Sylvie Ferrario-Méry, Christine H. Foyer, Jeremy Harbinson, Bertrand Hirel, Graham Noctor, Loïc Lepiniec, Anis M. Limami, Céline Masclaux-Daubresse, Jean-Claude Pernollet, Agnès Ricroch, Akira Suzuki

**Affiliations:** 1 Institute for Plant Sciences of Montpellier (IPSiM), Campus INRAE/Institut Agro. Bât. 7—2 Place Pierre Viala, 34060 Montpellier Cedex 2, France; jfbriat@free.fr; 2 INRAE, EVA, Université Caen-Normandie (UNICAEN), CEDEX, F-14032 Caen, France; jean-bernard.cliquet@unicaen.fr; 3 Laboratoire Génome et Développement des Plantes, UMR 5096 CNRS-Université de Perpignan, 66100 Perpignan, France; delseny@univ-perp.fr; 4 INRAE, AgroParisTech, Institute Jean-Pierre Bourgin for Plant Sciences (IJPB), Université Paris-Saclay, 78000 Versailles, France; sylvie.ferrario-mery@inrae.fr (S.F.-M.); loic.lepiniec@inrae.fr (L.L.); celine.masclaux-daubresse@inrae.fr (C.M.-D.); akirasuzuki004@gmail.com (A.S.); 5 School of Biosciences, University of Birmingham, Edgbaston B15 2TT, UK; c.h.foyer@bham.ac.uk; 6 Laboratory of Biophysics, Wageningen University, Stippeneng 4, 6708 WE Wageningen, The Netherlands; jeremy.harbinson@wur.nl; 7 Institute of Plant Sciences Paris-Saclay (IPS2), Université Paris-Saclay, CNRS, INRAE, Université Evry, 91190 Gif sur Yvette, France; graham.noctor@universite-paris-saclay.fr; 8 Institut de Recherche en Horticulture et Semences (IRHS), Université d’Angers, INRAE, 49000 Angers, France; anis.limami@univ-angers.fr; 9 Académie d’Agriculture de France, 18 rue de Bellechasse, 75007 Paris, France; jc.pernollet@icloud.com; 10 AgroParisTech, UFR Génétique, Évolutive et Amélioration des Plantes, 22, Place de l’Agronomie, 91120 Palaiseau, France; agnes.ricroch@agroparistech.fr

## 1. Jean- François Morot-Gaudry: The Man and the Scientist: Bertrand Hirel, Loïc Lepiniec, Céline Masclaux-Daubresse Recollections

It is with deep sadness that we learned of the passing of Dr. Jean-François Morot-Gaudry on 6 January 2024, after a long illness. Jean-François was a colleague and dear friend, known for his deeply human nature, honesty, warmth, and kindness. Born in 1943, he was a devoted husband, father of two, and proud grandfather of six.


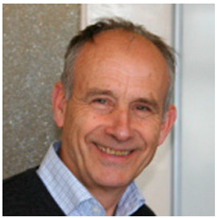


Jean-François was an Honorary Research Director at the “Intitut National de la Recherche pour l’Agriculture, l’Alimentation et l’Environnement” (INRAE; formerly INRA). He was elected Corresponding Member of the Academy of Agriculture in 1994 and later became a full member of the section titled Life Sciences in 2001. He was also a Knight of the National Order of Merit and a Commander of Agricultural Merit.

He graduated from the University of Dijon with a Bachelor’s degree in 1968, followed by a Master’s degree from the University of Montpellier in 1969. He then earned a “Diplome d’Etudes Approfondies” (DEA) in Plant Biology in 1970 and completed his PhD in 1979 at the University of Paris XI Orsay, under the supervision of Professor A. Moyse.

Jean-François joined INRAE Versailles in 1970 as a the “Centre National de la Recherche Scientifique” (CNRS) research member and became an INRAE research fellow in 1975, working in the laboratory of Plant Nutrition led by Eugène Jolivet and Yves Coïc. During his doctoral studies, he focused on photosynthesis and maize genetics. He later served as a visiting researcher at the “Comissariat à l’Energie Atomique” (CEA) Saclay and Cadarache (1977–1980) and spent several months in Canberra, a world-leading laboratory in photosynthesis and N metabolism, under the direction of B. Osmond in 1981.

Upon his return to INRAE, he played a key role in developing the laboratory of “Metabolisme et Nutrition des Plantes”, which he led for over fifteen years. Under his leadership, the laboratory fostered integrated research in physiology, biochemistry, molecular biology, and genetics, collaborating with numerous international researchers. He was promoted to Research Director in 1983 and to Director of Research (Exceptional Class) in 2004.

Jean-François’s research on photosynthesis and nitrogen (N) assimilation has been published in top international scientific journals. His expertise and deep understanding of plant nutrition, carbon, and N metabolism have also led to the publication of several authoritative reference works.

Jean-François also held significant administrative roles at INRAE, serving as Director of the Plant Metabolism and Nutrition Laboratory for 16 years and as Deputy Head of the Plant Biology Department for a total of 12 years (1986–1990 and 1995–2002). During this time, he initiated and led numerous fundamental and applied research projects in close collaboration with the scientists and technicians in his team. His research spanned a wide range of themes, from field experiments to the application of advanced molecular genetic techniques in plants.

In the applied research domain, his work included studies on vine coulure, the physiology of endive, innovative cropping systems involving nutrient recycling between fish farming and cultivated plants, and the impact of alga extracts on plant growth and development. His laboratory also pioneered the development of several cutting-edge analytical biochemistry techniques, leveraging his expertise in plant nutrition—particularly carbon and N metabolism. A notable achievement was his exploration of the genetic variability of both model and cultivated plants to deepen understanding of their physiology in relation to N use, a contribution that has left a lasting mark on plant biology at both national and international levels.

From 2008 to 2011, Jean-François served as an INRAE Project Manager. In addition to his research and administrative responsibilities, he was actively involved in teaching as an associate professor at the Universities of Versailles-Saint-Quentin (1994–2004) and Evry (1999–2000). He also authored around a dozen review works, including two books on plant biology (published by Dunod) intended for students at leading biology schools and universities.

Jean-François played a prominent role in the Academy, holding positions such as Secretary of the Life Sciences Section (2002–2008), Vice-Secretary of the Academy (2004–2009), Vice-President (2010), and President (2011). He was a member of numerous academic (2004–2015) and communications (2009–2012) committees, organized many public sessions between 2001 and 2012, and authored some twenty publications for the Academy.

A moving biography was published in *Archives Contemporaines* and is available in French (Jean-Francois Morot-Gaudry. Archorales: les métiers de la recherche, témoignages, 13, Editions INRA, 159 p., 2008, Archorales, 978-2-7380-1258-6).

During the memorial event held at INRAE Versailles on 1 October 2025, scientists who worked in his unit retraced the history and scientific impact of the research conducted in Jean-François Morot-Gaudry’s laboratory, work that remains highly relevant in the field of plant biology today.

In the following section of this introductory chapter, several members of Morot-Gaudry’s laboratory, along with other scientists, pay tribute to Jean-François. They reflect on how science and scientific administration were conducted under his leadership and highlight the major achievements that emerged from his laboratory.

## 2. Jean-François Briat Recollections

Among his numerous professional activities, Jean-François had a major role in the management of the Plant Biology department from INRAE. It was a small (about 170 permanent staff scientists, and technicians), atypical department, with a high scientific standard, very much in line with the biology laboratories of the CNRS and the CEA, some of INRAE department laboratories being in fact also formally associated with the CNRS and/or the CEA. This department had always been recognized by its peers, both nationally and internationally. Until the late nineties the successive department heads were primarily responsible for coordinating the initiatives of each laboratory. In 2013, this department fused with the Genetic and Breeding department to constitute the present Biology and Plant Breeding department which is the biggest INRAE department.

When Guy Paillotin took over as executive head of INRA in 1987, Roland Douce, a professor at the University of Grenoble, was appointed head of the Plant Biology department. He was assisted by Jean-Claude Pernollet and Jean-François Morot-Gaudry, both of whom helped manage the department for four years.

At that time, leading and managing an INRAE department was a fairly easy task since the departments were not autonomous and the important decisions were taken by the scientific directors. The main activity of the department heads consisted of setting the broad research directions and handling the recruitment and competitive examinations for researchers and engineers. This remained a very scientific job, and for a small department such as the Plant Biology Department, it was straightforward and not too time consuming to be achieved.

From 1990 to 1995, Jean-François was no longer assistant of the head of the department; he devoted this “period of freedom” as he said himself to his own research, in particular through fruitful collaborations with the University of North Carolina at Raleigh (USA), where I met him for the first time. However, in 1995, Alain Pradet from INRAE Bordeaux, and new head of the Plant Biology department, appointed him again as deputy head. After the retirement of Alain Pradet, Jean-François retained this position of deputy, supporting Christian Dumas, a professor at the “École Normale Supérieure” (ENS) at Lyon, who became the new head of the Plant Biology department.

This was a pivotal period because the new director of INRAE, Paul Vialle, had initiated a profound reform of the organization and management of the Institute. Jean-François was very involved in this reform and actively participated in the analysis of the major INRAE survey that preceded it. In three weeks at the end of 1997, some fifteen INRAE staff members (among them Jean-François) analyzed the results of surveys on the Institute’s operations conducted at the departmental and research center levels. Based on this survey, Paul Vialle launched his reform in 1997, one of the principal aims being to give autonomy to the departments. This had a positive side, with more freedom granted to departments, but also imposed to took over all administrative, financial, and human resources management, as well as contract and patent management.

However, not so much was achieved in that way by the Plant Biology department between 1997 and 1999, and when I replaced Christian Dumas as head of the Plant Biology department in 1999, I discovered that the reform that was already deeply advanced in other departments was at a standstill in the Plant Biology department, and that we had to face a major challenge in a very short time. We had to produce a strategic plan for the four years ahead, this document being the framework for discussions with the INRAE scientific director, Guy Riba, in order to negotiate the required resources to conduct the plan. Jean-François was of an incredible help during this period. He was the memory of the department and his analytical finesse and his knowledge of all the mysteries of INRAE prevented me from getting lost in many aspects. Jean-François was incredibly efficient in helping me to set this new organization of the department. At the end of our four years together, the Plant Biology department was reorganized and structured according to the attempt of the scientific direction. In 2002 we conducted the first evaluation of the department which was also very demanding and for which Jean-François was once more the perfect collaborator.

After 2002, Jean-François no longer held any official positions in the department of Plant Biology, which he therefore served with great dedication under four successive heads of department for 12 years. He was its living memory, not only on a scientific level but also through his very human knowledge of all the staff, whom he called often by their first names and whose professional background he knew, but often their life stories. Jean-François was driven by an exceptional sense of community and had a rare sincerity in his attention to others.

## 3. Jean Bernard Cliquet Recollections

I completed my master’s project in Jean-François Morot-Gaudry’s laboratory, though I didn’t work directly with him. At the end of the project, he successfully secured a PhD grant for me, a brilliant move on his part. He took me to meet the scientific director of a small agrochemical company on a hot Friday evening in summer. Even 40 years later, I still vividly remember Jean-François sketching the leaf carbon cycle, focusing on photorespiration, on a whiteboard while the director watched in awe. That is how I obtained a CIFRE (Conversion Industrielle de Formation de Recherche) grant.

The actual thesis topic, however, had nothing to do with that initial meeting. Instead, it focused on the effects of a stem shortener applied to corn, which was reducing yield. Jean-François suggested using stable isotopes (^13^C and ^15^N) to understand why the product was affecting yield, and he introduced me to Eliane Deleens, France’s leading expert in ^13^C isotopes. This was another stroke of genius, as Eliane and I worked exceptionally well together. She was a remarkable person, and we accomplished great work as a team. Sadly, she passed away a few years after I completed my PhD.

Officially, Jean-François was my supervisor, but when it came time to assemble my PhD defense committee, he instructed me to list Eliane as my thesis director. He chose to serve as a reviewer alongside Eliane’s boss, Jean-Louis Prioul. Of course, such an arrangement would be unthinkable today. Ironically, his review was relatively harsh. Before that, he had meticulously corrected and improved the papers we published during my PhD. There is one particular story about him and Jean-Louis Prioul that I always share when discussing authorship with colleagues.

I had my first paper published in *Plant Physiology*. I was the first author, but there were quite a few co-authors because we wanted to include the technical staff and the director of the lab where we conducted the isotopic analyses. Jean-François and Jean-Louis decided it would be better for me not to have too many authors on my second paper in *Plant Physiology*. Even though they had contributed to the work, they removed their names at the last moment. I don’t often see people so selfless and dedicated. Jean-François was not only a great scientist and leader, but also an incredible human being.

## 4. Michel Delseny Recollections

I first met Jean-François Morot-Gaudry in the mid-1980s during a visit to Versailles. Although our research interests differed, our shared Burgundian roots brought us closer, and we quickly became friends. I made numerous visits to the INRAE center in Versailles for thesis defenses, seminars, or to discuss projects and results with my colleagues Jacques Tempé, Francine Casse, Michel Caboche, Georges Pelletier, and many others. On each of these visits, I always made sure to see Jean-François and exchange a few words about current scientific events and our projects. I would like to highlight three occasions during which we interacted, and through which I came to appreciate his numerous qualities.

The first concerns his publishing activities. I was delighted to see the publication of his two-volume work, *Plant Biology, Growth and Development (Volume 1) and Nutrition and Metabolisms (Volume 2)*, co-authored with Roger Prat. These volumes arrived at a particularly opportune time, updating our knowledge in the field. This work, enriched through three or four successive editions, served as a bible for many students, teachers, and researchers. It reflects Jean-François’s passion for sharing knowledge and his talent for teaching. He applied this commitment to disseminating knowledge not only within his own research area, producing reference works on N nutrition and photosynthesis, but also in emerging fields by bringing together specialists. This is how he coordinated *Genomics in Plant Biology* with Jean-François Briat, published in 2004. I was honored when he asked me to contribute two chapters and to share ideas about the future of this discipline. The book was a resounding success, translated into English in 2007 with the help of his friend Peter Lea, and even into Chinese the following year, a virtually unique achievement in the history of INRAE.

The second opportunity I had to work with him was my participation in the Foresight Workshop “What Sustainable Plants and Production Systems for Biomass in the Future”, organized by INRAE in 2009 and coordinated by Paul Colonna. This workshop was a unique chance to collaborate with Jean-François. He led the working group tasked with identifying plants containing potentially interesting chemical structures. I learned a great deal from him and gained a deep appreciation for the breadth of his scientific knowledge. The experience was exhilarating, and we felt we were laying the groundwork for the adventure of green chemistry. The few meetings we had, followed by email exchanges, were fascinating and took place in a friendly, open atmosphere, largely thanks to Jean-François’s qualities and the rigor with which he led discussions and assigned tasks. His role was not limited to leading his own working group as he also participated in four others. This broad engagement allowed him to guide his group in a particularly dynamic and insightful way, reflecting both his intellectual curiosity and his desire to prepare for the future on a scientific basis.

The final point I wish to mention is the role he played in fostering the relationship between the French Academy of Agriculture (AAF) and the Academy of Sciences. He was elected a corresponding member of the AAF in 1994 and a full member in 2001. He was deeply involved in the life of the Academy, taking on many thankless tasks and even serving as its President. In this capacity, he organized numerous scientific meetings, including several joint events with the Academy of Sciences, on topics such as flowers, plant biotechnologies, vegetative development, the plant cell wall, innate immunity in plants, species domestication and migration, and genetically modified organisms (GMOs). He also participated in the tributes paid at the Academy of Sciences to Roland Douce and Michel Caboche. His work demonstrates his commitment to sharing the most up-to-date knowledge and his tireless efforts to bring cohesion to communities whose interests sometimes diverged but were united by a common foundation of scientific excellence.

I remember him as an outstanding colleague and friend, extremely kind, open-minded, and deeply committed to the public good. He was imbued with a collective spirit and devoted to his community.

## 5. Sylvie Ferrario-Méry Recollections

My first memory of Jean-François dates back to the 1980s, when he visited the INRAE Agronomic Station in Antibes. At the time, I was a doctoral student and later a young researcher there. Jean-François was the director of the Plant Metabolism and Mineral N utrition Laboratory at INRAE in Versailles. He showed a keen interest in the research conducted in Antibes, as it shared similarities with the research themes of the Mineral Nutrition Laboratory in Versailles. He also presented the main research themes of his own laboratory.

Later, when I requested a transfer to a research laboratory at INRAE in Versailles, it felt natural to reach out to Jean-François. In 1994, he welcomed me warmly, while respecting the hierarchy, of course. In his laboratory, I initially worked with Christine Foyer until she moved to Aberystwyth in Wales. Shortly afterward, I continued collaborating with her. I also met former colleagues Bertrand Hirel and Akira Suzuki there, with whom I had begun my research in Prof. Pierre Gadal’s Laboratory in Orsay during my master’s studies.

At that time, I was developing research on the interactions between carbon and N metabolism in plants—exciting topics that allowed me to build connections and exchange ideas with the different members of the laboratory. This collaboration gave me the opportunity to participate in various European research projects with my colleagues.

I will always be grateful to Jean-François for welcoming me into his Laboratory of “Metabolisme et Nutrition des Plantes” at the INRAE Center in Versailles. This opportunity allowed me to develop my research career within the prestigious INRAE Center in Versailles at the start of my professional journey.

## 6. Christine Foyer Recollections

I was fortunate to join the “Laboratoire du Métabolisme et de la Nutrition des Plantes” in Versailles in 1988. I was encouraged to apply for the position of Directeur de Recherche by Professor Roland Douce, who spoke highly of the laboratory. After submitting my application, Jean-François reached out to me, and I made several visits to Versailles before officially taking up the post.

From the very beginning, Jean-François and the entire lab were incredibly welcoming. I quickly settled into my work in the team, despite my limited French and the fact that most of the team, apart from Jean-François, spoke little English.

During my first months in Versailles, I shared an office with Jean-François. I soon discovered that he was a dynamic colleague who took great pleasure in his administrative responsibilities in running the lab. His ability to support, empower, and mentor researchers was matched by his fairness and exceptional organizational skills. Above all, he was deeply passionate about plant science, particularly photosynthesis. While he was always fully supportive of innovative basic research, he remained mindful of the needs of agriculture, especially the impact of environmental stress on the production of grapes and other key crops. He was an outstanding role model, and his positive attitude toward both the study and application of science was truly infectious.

Jean-François was always open to collaborations and welcomed several international scientists into the lab. For instance, Jeremy Harbinson, from the UK and later the Netherlands, visited multiple times over several years. He brought his custom-built fluorescence imaging systems, which included a freeze-clamping mechanism to preserve the metabolic state of leaves. This allowed us to measure changes in key photosynthetic metabolites and enzyme activation states. The lab’s output of high-quality scientific papers is a testament to Jean-François’s ambition, leadership, and competent management. He made the day-to-day work in the lab both enjoyable and deeply rewarding.

During my time in Versailles, I published over sixty papers and continued collaborating with colleagues there long after I returned to the UK. I look back on my time working with Jean-François with deep affection. He was not only a kind and considerate colleague and manager but also a consummate diplomat, handling interactions at every level with good humor, excellent communication, and emotional intelligence.

I also learned to appreciate fine red wine and to understand the true meaning of “joie de vivre”, qualities that Jean-François embodied. His cheerful, confident enjoyment of life and his positive, exultant spirit enriched everyone who knew him.

## 7. Jeremy Harbinson Recollections

Jean-François was a very cheerful, welcoming, and modest man who always seemed genuinely happy to see me. He was unfailingly positive about my attempts at schoolboy French, despite my rather heavy Belfast accent. Jean-François also showed great interest in the somewhat makeshift machines that Christine and I used for our photosynthetic measurements.

I remember how his good-natured attitude extended to the entire group of “Laboratoire du Métabolisme et de la Nutrition des Plantes” which was a friendly and supportive environment, filled with people who genuinely cared. When I look back on my time in the Versailles lab, I do so with great happiness, and I believe this warmth came from Jean-François himself. His kindness and generosity created a welcoming atmosphere for everyone in the group.

## 8. Anis Limami Recollections

I worked for twelve years with Jean-François Morot-Gaudry at the laboratory of “Metabolism et Nutrition des Plantes”, a research unit affiliated with INRAE. My research focused on chicory and was partially funded by the French professional organization called “Fédération Nationale des Producteurs d’Endives” (FNPE), which supported several PhD projects. My work investigated the effects of mineral nutrition on various physiological and metabolic aspects related to plant growth and the quality of the harvested organ, the edible shoot, known as the chicon. Initially, I studied calcium nutrition, particularly calcium mobility within the plant, aiming to improve calcium delivery to the fast-growing chicon. This was intended to prevent localized deficiencies and the appearance of black spots (tissue necrosis), which negatively impact commercial quality. A significant part of my research, conducted in collaboration with Jean-François, examined how nitrate supply affects the growth, development, and disease susceptibility of chicory-endive, with a focus on how these effects vary depending on the plant genotype. Below, I summarize the major findings of this work, particularly the contributions of Dr. Céline Richard-Molard.

Chicory-endive is a biennial plant that forms a rosette and a tuberized root during its first year. The root stores carbon and N reserves, which are mobilized during a subsequent forcing phase under controlled environmental conditions to produce the edible shoot, the chicon. This unique growth cycle provides an opportunity to investigate how nitrate supply during the initial growth phase influences later shoot development and disease resistance, potentially through internal signaling mechanisms.

Increasing nitrate supply during the vegetative phase stimulated shoot growth without significantly affecting tuberized root biomass, thereby raising the shoot-to-root ratio. Additionally, chicons produced by plants grown under higher nitrate conditions exhibited greater susceptibility to soft rot, a bacterial disease.

To investigate whether sensitivity to high nitrate nutrition is genetically determined, we compared the responses of several homozygous lines subjected to either low or high nitrate supply during the vegetative phase. We then analyzed the growth and quality of their chicons produced during the forcing phase. The results revealed a genotype-dependent response, allowing us to distinguish two groups of homozygous lines: nitrate-sensitive (NS) and nitrate-resistant (NR). Genetic clustering, based on a DNA fingerprinting method for calculating genetic distances, confirmed the classification of the analyzed homozygous lines into the two distinct groups: NS and NR.

The observation that nitrate nutrition during the vegetative phase determines both chicon morphology and susceptibility to bacterial disease suggests the existence of a signaling mechanism mediating the effects of nitrate supply. This signal may be stored in the root during the first year of growth and subsequently transmitted to the chicon during the forcing period.

Since root carbon compounds remained stable across genotypes and nitrate treatments, they were deemed unlikely to serve as signaling agents. In contrast, N compounds, including total nitrogen, nitrate, and amino acids—varied between genotypes depending on nitrate levels. Notably, nitrate-sensitive (NS) lines exhibited lower N content compared to nitrate-resistant (NR) lines. Amino acid profiling further revealed substantial differences: NR lines accumulated higher levels of glutamine, while NS lines accumulated more glutamate, indicating divergent N storage and metabolic strategies.

This original work expands our understanding of nutrition, not only as an essential nutrient but also as a critical signaling factor in plants. The findings have significant implications for both crop management and breeding strategies, particularly in optimizing morphological and thus commercial quality and disease resistance in chicory-endive, and potentially in other crop species as well.

## 9. Graham Noctor Recollections

My memories of Jean-François’s character and personality resonate deeply with those of Christine Foyer. He was an exceptionally kind and generous man, who had a unique talent for making everyone feel welcome in the laboratory. I joined the “Laboratoire du Métabolisme et de la Nutrition des Plantes” in Versailles in 1994 as a post-doc, working on a project led by Christine in collaboration with Lise Jouanin from the laboratory of “Biologie Cellulaire” located upstairs. Thanks in no small part to Jean-François, my time in Versailles proved to be incredibly productive and rewarding. I was honored to co-author two papers with him in the 1990s, though I must admit my contributions were largely limited to language editing rather than scientific content.

One of the things I remember most vividly is how encouraging he was as I struggled to learn French. Jean-François was a patient and enthusiastic teacher, and I learned so much from him, especially during our many engaging Friday evening discussions. We explored the similarities and nuances between French and English, delved into the history of both languages, and shared insights into French culture. His passion for these topics made every conversation both informative and inspiring.

Jean-François was also deeply committed to supporting the careers of those around him. He played a pivotal role in securing an INRAE fellowship for me, which allowed me to continue my research after my initial European Union (EU) funding ended. It was also Jean-François who first suggested I prepare a dossier for the “Habilitation à Diriger des Recherches” (HDR), with the goal of applying for a professor position in France. He introduced me to Pierre Gadal, then director of the plant sciences doctoral school, and thanks to his guidance, I obtained the qualification the year after leaving INRAE. His influence on my career was profound and lasting, and for that, I will always be immensely grateful.

Working with Jean-François was a true pleasure, and it was a privilege to have known him and learned from him. His kindness, generosity, and intellectual curiosity left a lasting impression on everyone who had the chance to cross his path.

## 10. Jean-Claude Pernollet Recollections

It is with profound emotion that I reflect on a friendship that has endured for nearly half a century. I first met Jean-François 48 years ago, upon my arrival at INRAE in Versailles, where our laboratories were neighbors. What began as a simple proximity soon blossomed into a collaboration and, ultimately, a deep and lasting friendship. For nearly fifty years, we walked parallel paths, not only at INRAE but also within the French Academy of Agriculture, where Jean-François made an indelible mark through decades of dedicated service. Our journey together began in early 1977, when Jean-François was still pursuing his Doctorat ès Sciences, splitting his time between the CEA facilities in Saclay and Cadarache and the “La Minière” INRAE laboratory. His research focused on the distinctive features of C_4_ metabolism in maize, particularly in the opaque2 mutant, a fascinating variant known for its elevated levels of essential amino acids, though at the cost of reduced yield. Our early careers unfolded in parallel, both rooted in university laboratories outside the INRAE center. This shared experience naturally drew us closer, leading to a fruitful collaboration. Together, we explored the assimilation of atmospheric CO_2_, tracing its path to the storage proteins in maize kernels.

As laboratory heads, our responsibilities brought us even closer. We both served as assistants to Professor Roland Douce, Head of the Plant Physiology Department, whose laboratory was based at the CEA in Grenoble. In this role, we acted as Roland’s “right hands” managing the day-to-day operations of the department while he focused on major scientific issues. For four demanding years, we balanced these administrative duties with the leadership of our own laboratories, a dual responsibility that required unwavering commitment.

Jean-François was a pivotal figure in education, playing a key role in establishing a Master’s degree in Biology at the University of Versailles Saint-Quentin after its independence from the University of Paris VI in 1994. He taught extensively, devoting nearly a third of his time to education, not only in Versailles but also at the University of Évry and through contributions to Master’s programs, particularly at the University of Paris VI. His commitment to scientific publishing was equally remarkable. He authored around ten major synthesis works for researchers and students, including two textbooks on plant biology designed for students in leading biology schools and universities. Beyond academia, he dedicated himself to science communication, contributing to public exhibitions, notably at the “Cité des Sciences et de l’Industrie” located in Paris.

Though I had moved to another INRAE research center at Jouy-en-Josas in 1995, our professional paths re-converged through the French Academy of Agriculture. At the Academy, Jean-François embraced new intellectual horizons, particularly in the economic and societal dimensions of agricultural science. He presided over countless weekly sessions, often in collaboration with the Academy of Sciences, and his interpersonal skills made him a natural steward of scientific programming. He meticulously reviewed every application file submitted to the Academy and participated in numerous working groups, always bringing clarity and commitment.

Jean-François was more than a colleague, he was a loyal friend to the very end. His humanity, honesty, warmth, and benevolence left an indelible mark on everyone who knew him. He remained an inexhaustible worker, whether in the laboratory, at home, or even in his later years, when he was still felling trees at his Britany retreat at the age of 80.

Endowed with a vast scientific culture, Jean-François had a gift for inspiring curiosity, especially in his grandchildren, to whom he explained the wonders of nature with kindness and patience. His passion for sharing knowledge led him to publish not only scientific articles but also books for students and the general public, a testament to his generosity.

Jean-François’s passing leaves an immense void, not only in the scientific world, where he was a living encyclopedia of plant metabolism and photosynthesis, but above all, on a human level. His warmth, his welcoming nature, and his unwavering kindness defined every relationship he nurtured.

## 11. Agnès Ricroch Recollections

At the “Académie d’Agriculture de France”, Jean-François Morot-Gaudry was elected as a corresponding member in 1994 and later as a full member in 2001. Throughout his tenure, he held numerous voluntary positions, generously dedicating his time and expertise to the institution.

Through the sessions and symposia, he organized or co-organized, Jean-François shared his profound knowledge of plant biology and physiology with a broad and engaged audience. His public sessions spanned a diverse range of topics, from fundamental plant biology to its technological and agronomic applications, making complex science accessible to all.

A scholar of remarkable scientific culture, Jean-François authored multiple books for students of plant sciences, distilling his expertise into clear and insightful resources.

As Secretary of the Life Sciences Section from 2017 to 2025, I had the privilege of benefiting from his deep insights into the life sciences. We collaborated closely within the Academic Commission and the Program Commission, which organizes sessions and symposia for the section’s members throughout the year. The Life Sciences Section, comprising 76 scientists and engineers, explores major questions spanning the plant, animal, and microbial worlds, from molecules to ecosystems. By combining mechanistic and integrative approaches, the section seeks to understand and model the biological processes that define the unity and diversity of life.

Jean-François was an affable and gifted communicator, able to explain the complex mechanisms of plant physiology, the focus of his research career, with clarity and simplicity. His intellect and wisdom supported me throughout my three terms in leadership roles within the section. He fostered a collegial atmosphere, where I prioritized freedom of expression and courteous debate as cornerstones of our discussions.

As a former President of the Academy, Jean-François worked alongside me in the Academic Commission, always acting for the common good of the section and the broader institution.

Through his books and teachings, Jean-François Morot-Gaudry remains an exemplary scientist, one who examined complex living processes with simplicity and elegance. His work continues to inspire our reflections, leaving a lasting legacy in both science and mentorship.

## 12. Akira Suzuki Recollections

The Sixth International Congress on Photosynthesis was held from 1 to 6 August 1983 on the campus of the Vrije Universiteit Brussel in Brussels, Belgium. Jean-François Morot-Gaudry attended this landmark event, as did members of our laboratory from Orsay, who were present independently. At the time, the Orsay group belonged to the Laboratory of Plant Metabolic Physiology at the University of Paris XI. I was a student in the Doctorat d’État program, and our research focused on two key areas of plant science: carbon fixation and N assimilation into amino acids.

At the close of the congress, during one of the evening discussions, Jean-François and the Orsay group found themselves sharing a table at a restaurant near the campus. It was there that I introduced myself to him for the first time. By then, Jean-François was co-directing the Laboratory of Nutrition at INRAE Versailles, having recently completed a seven-month postdoctoral stay in Professor Barry Osmond’s laboratory in Canberra, Australia between 1981 and 1982.

In 1986, I joined the INRAE Versailles center as a research scientist after passing the entrance examinations. Jean-François became the head of the Laboratory of “Metabolisme et Nutrition des Plantes” in 1987, and it was in this lab that I conducted my research. Despite his administrative responsibilities, he occasionally returned to the bench, conducting experiments on photosynthesis using ^13^CO_2_/^14^CO_2_ fixation with his custom-built device, though his hands-on work gradually diminished over time.

The 1980s marked a turning point in plant science, as emerging molecular biology techniques and reverse genetics began to unravel the fundamental mechanisms of inorganic N assimilation in plants. One of my research projects focused on characterizing the structure of glutamate synthase (GOGAT) and its role in regulating amino acid synthesis during primary nitrate reduction and photorespiration.

In March 1990, I began a collaboration with Professor S. Rothstein at the University of Guelph, Ontario, Canada. At that time, the Arabidopsis Genome Initiative had not yet been established, and our work relied on a single published nucleotide sequence of the *E. coli* NADPH-glutamate synthase gene (1987). Using cDNA and genomic clone screening, we identified a sequence of Arabidopsis ferredoxin-dependent GOGAT (Fd-GOGAT).

Our experiments also revealed the presence of a second Fd-glutamate synthase gene through genomic Southern blotting, as well as tissue-specific and light-responsive expression patterns. This eighteen-month collaboration in Guelph equipped me with the expertise to further investigate ammonia assimilatory pathways upon my return to INRAE Versailles in September 1991.

My time in Guelph was also marked by frequent exchanges with Jean-François. During that period, INRAE conducted biennial evaluations of its researchers, and my assessment in 1991 coincided with my stay in Canada. We exchanged multiple drafts by fax (as email was not yet in use) to finalize my evaluation file, allowing Jean-François to stay updated on my progress in Professor Rothstein’s lab. He also sent me handwritten notes with updates on lab news, administrative requests, and his plans, including an upcoming trip to Raleigh, USA, in 1991 to visit a former colleague at North Carolina State University.

Jean-François cherished these overseas visits, which provided him with opportunities to return to bench work, particularly using ^15^N stable isotopes to study plant mineral nutrition. Despite his growing administrative duties, photosynthesis remained his passion. He was one of the organizers of the Xth International Photosynthesis Congress, held in Montpellier in August 1995.

Reflecting on those years, Jean-François often remarked that the peak of the lab’s scientific activity occurred between 1988 and 1995, a period marked by the diverse research strategies of his colleagues. I am proud to have contributed to this significant period as part of his team.

In 1999, the lab was reorganized into a new research unit, the “Laboratoire de Nutrition Azotée des Plantes”, with a focused mission on nitrogen-related plant sciences. One of the highlights of our international collaborations was the bilateral symposium in Nara, Japan in 2002, where ten colleagues from INRAE Versailles, including Jean-François, participated alongside twenty-three invited researchers.

This gathering reminded me of the Photosynthesis Congress in Brussels nearly twenty years earlier, reinforcing the enduring bonds of our scientific community. The bilateral programs, supported by CNRS, INRAE, CEA, (Japan Society for Promotion of Science (JSPS), and the Embassy of France in Tokyo, became a cornerstone of our long-term collaborations with the Japanese plant science community. These initiatives began with a symposium at INRAE Versailles in 1999 and continue to this day, with the upcoming IRN Symposium at CNRS Strasbourg in 2025.

Despite his extensive administrative and managerial responsibilities, Jean-François always considered himself, above all, a researcher who loved hands-on experimentation. He often described himself as “manual”, someone who thrived in the lab. Beyond research, he was a dedicated educator, teaching plant biology to students at multiple universities throughout the 1990s and 2000s. His commitment to teaching and research was inseparable, reflecting his belief that true scientific progress emerges from the synergy of discovery and mentorship.

His legacy endures not only in the scientific advancements he contributed to but also in the generations of researchers he inspired, including myself.

